# Deciphering Metabolic Adaptability of Leukemic Stem Cells

**DOI:** 10.3389/fonc.2022.846149

**Published:** 2022-06-08

**Authors:** Sweta B. Patel, Travis Nemkov, Angelo D’Alessandro, Robert S. Welner

**Affiliations:** ^1^ Department of Medicine, Division of Hematology/Oncology, O’Neal Comprehensive Cancer Center, University of Alabama at, Birmingham, AL, United States; ^2^ Divison of Hematology, University of Colorado Anschutz Medical Campus, Aurora, CO, United States; ^3^ Department of Biochemistry and Molecular Genetics, University of Colorado Anschutz Medical Campus, Aurora, CO, United States

**Keywords:** hematopoietic stem cells, metabolism, metabolic techniques, leukemic stem cells (LSCs), leukemia

## Abstract

Therapeutic targeting of leukemic stem cells is widely studied to control leukemia. An emerging approach gaining popularity is altering metabolism as a potential therapeutic opportunity. Studies have been carried out on hematopoietic and leukemic stem cells to identify vulnerable pathways without impacting the non-transformed, healthy counterparts. While many metabolic studies have been conducted using stem cells, most have been carried out *in vitro* or on a larger population of progenitor cells due to challenges imposed by the low frequency of stem cells found *in vivo*. This creates artifacts in the studies carried out, making it difficult to interpret and correlate the findings to stem cells directly. This review discusses the metabolic difference seen between hematopoietic stem cells and leukemic stem cells across different leukemic models. Moreover, we also shed light on the advancements of metabolic techniques and current limitations and areas for additional research of the field to study stem cell metabolism.

## Introduction

Cancer cells metabolically reprogram to thrive in a stressed environment, lead to disease progression and even drug resistance. This is considered as metabolic adaptability of cancer cells. They can be metabolically flexible (ability to use different substrates as a source of energy) and/or metabolically plastic (ability to process a substrate through different pathways). Although metabolic adaptability is beneficial for the cancer cells, it can also serve as a limiting factor for their survival and hence making them vulnerable to inhibitors. This review will focus on such metabolic adaptations seen in leukemic stem cells compared to healthy counter parts as well as advancement and limitation in the field (1).

Like hematopoietic stem cells (HSCs), leukemic stem cells (LSCs) are quiescent, have self-renewal potential and the ability to engraft. Additionally, LSCs are also defined by their leukemogenic potential. To eradicate LSCs, researchers are exploring the field of metabolism to find metabolic differences between LSCs and their healthy counterparts. In leukemia, transformed cells acquire metabolic changes, which could be oncogene driven in addition to environmental or stress cues to aid survival. Not only do LSCs in the bone marrow metabolize differently from non-transformed cells, but they also have a different metabolic profile from the bulk of the disease. Furthermore, LSCs are a heterogeneous population and, in some cases, rare cells, posing a limitation to study. However, with an increase in the number of studies related to metabolism, new techniques have been developed to overcome the shortcomings of older methods. In this review, we focus on the metabolic difference between hematopoietic and leukemic stem cells, the new techniques developed and the challenges in studying and metabolically targeting leukemic stem cells.

## Metabolism of Hematopoietic Stem Cells

HSCs are found in a hypoxic environment of the bone marrow (2). HIF-1α (Hypoxia Inducible Factor) is a hypoxic sensor stabilized under low oxygen concentration (3). Among other genes, HIF-1α induces expression of lactate dehydrogenase (LDH) and pyruvate dehydrogenase kinase (PDK) gene family, which are essential regulators of anerobic glycolysis (4). Inhibition or deletion of HIF-1α, PDK2/4, and LDHA leads to increased proliferation and exhaustion of the HSCs (4–6). However, inhibition of pyruvate kinase (PKM2), another glycolysis rate-limiting enzyme, has no impact on the regular function of HSCs unless they are stressed by serial transplant (4). On top of glucose metabolism, fatty acid desaturation (FADS) and oxidation (FAO) are also essential for the maintenance, proliferation, and differentiation of HSCs (7–9). An active FAO leads to asymmetric cell division of HSCs giving rise to downstream progenitors, while inhibition of FAO leads to symmetric cell division, promoting self-renewal (8).

Owing to the glycolytic nature of HSCs, compared to the hematopoietic progenitors and mature hematopoietic cells, they have reduced mitochondrial respiratory capacity, turnover rate, and mitochondrial activity but a high mitochondrial mass (10, 11). Inhibition of mitochondrial carrier homolog 2 (MTCH2), a regulator of mitochondrial activity, leads to an increase in mitochondrial size, ATP production, and reactive oxygen species (ROS), leading to a shift from glycolysis to oxidative phosphorylation (OxPhos) that triggers the entry of HSCs into cell cycle (12). This subsequently causes a functional decline of HSCs, marked by the accumulation of dysfunctional mitochondria due to asymmetric cell division and loss of Dynamin-related protein 1 (Drp1) function, a key regulator of mitochondrial fission (13). On the contrary, mitochondrial fusion protein, mitofusin2 contributes to maintaining lymphoid potential in HSCs (14). Moreover, inhibiting fumarate hydratase, a critical enzyme of the citric acid cycle (CAC), reduces the long-term repopulating potential of HSCs (15). On the contrary, inhibiting mitochondrial phosphatase, Ptpmt1, impairs HSC differentiation by activating 5’ Adenosine Monophosphate-activated Protein Kinase (AMPK), aiding in the maintenance of self-renewal potential (16, 17). Individual metabolites also play an important role in maintaining of HSCs (18); however, significantly more work needs to be done to understand their role in HSC function.

**Figure 1:**
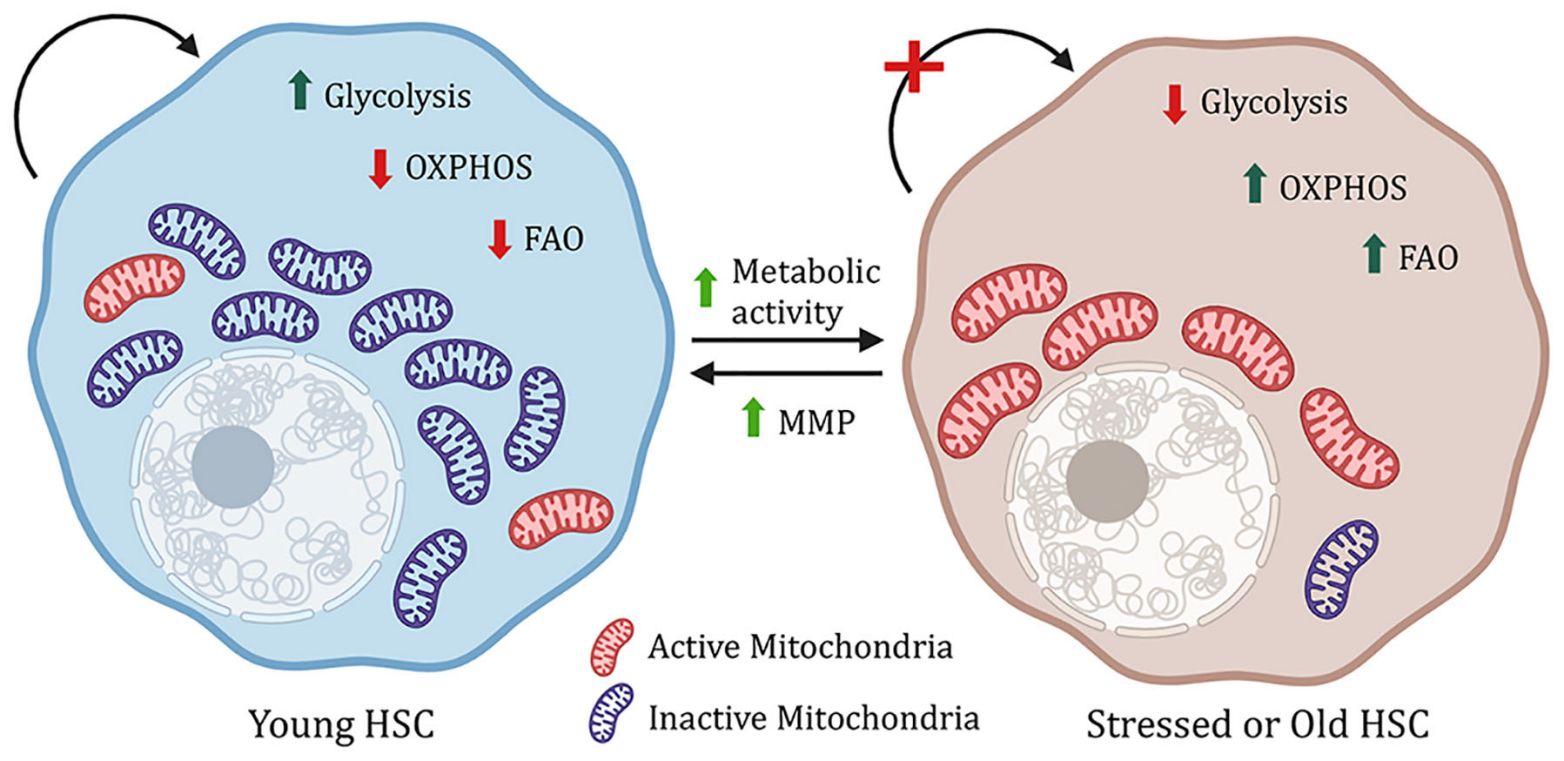
Metabolic regulation of HSCs. Young hematopoitic stem cells have a higher mitochondrial mass, but the mitochondria are inactive, and the cells rely on glycolysis. However, under stress, increase in metabolic activity, OXPHOS (oxidative phosphorylation) and FAO (fatty acid oxidation) lead to impaired stem cell function. This phenomenon can be reversed by increasing mitochondrial membrane potential (MMP).

Most of the data collected above were done with young HSCs, however, opposed to young HSCs, aged HSCs have increased autophagy, dysfunctional chaperone-mediated autophagy, metabolic activity, myeloid bias with reduced engraftment, and hematopoiesis potential, as well as accumulation of dysfunctional mitochondria (9, 13, 19). However, increasing the mitochondrial membrane potential using mitochondrially targeted coenzyme-Q10 (Mitoquinol, MitoQ) or pharmacological activation of chaperon mediating autophagy reverses the aging HSC phenotype (9, 20). This indicates that the right balance of anerobic glycolysis and OxPhos is essential for maintaining healthy HSCs (**Figure 1**).

## Metabolism in Myeloid Leukemia

### Acute Myeloid Leukemia (AML)

AML is mostly a clonal disorder commonly seen in the aged population and comprises 62% of all leukemia-related deaths (21). It is genetically heterogeneous and complex leukemia with multiple known mutations (NPM1, CEBPa, DNMT3A, Flt3-ITD, IDH, TET2, ASXL, SF3B1) and translocations (AML-ETO, MLL-AF9) (22). Patients usually have more than one of these cytogenetic abnormalities, and based on the combination, a patient’s prognosis can be predicted (23, 24). AML LSCs are metabolically classified as cells with low levels of ROS. LSCs with less ROS are quiescent, have a self-renewal potential, and resist drug treatment (25). These cells are dependent on mitochondrial function as well as mitophagy for their survival. Inhibition of mitochondrial translation, mitochondrial chaperonin, CLPB or even mitophagy regulator, FIS1 (Mitochondrial Fission 1 protein) and its upstream target AMPK leads to loss of LSC self-renewal potential, myeloid differentiation, and cell cycle arrest and ultimately cell death even for the resistant AML LSCs (26–28).

**Figure 2:**
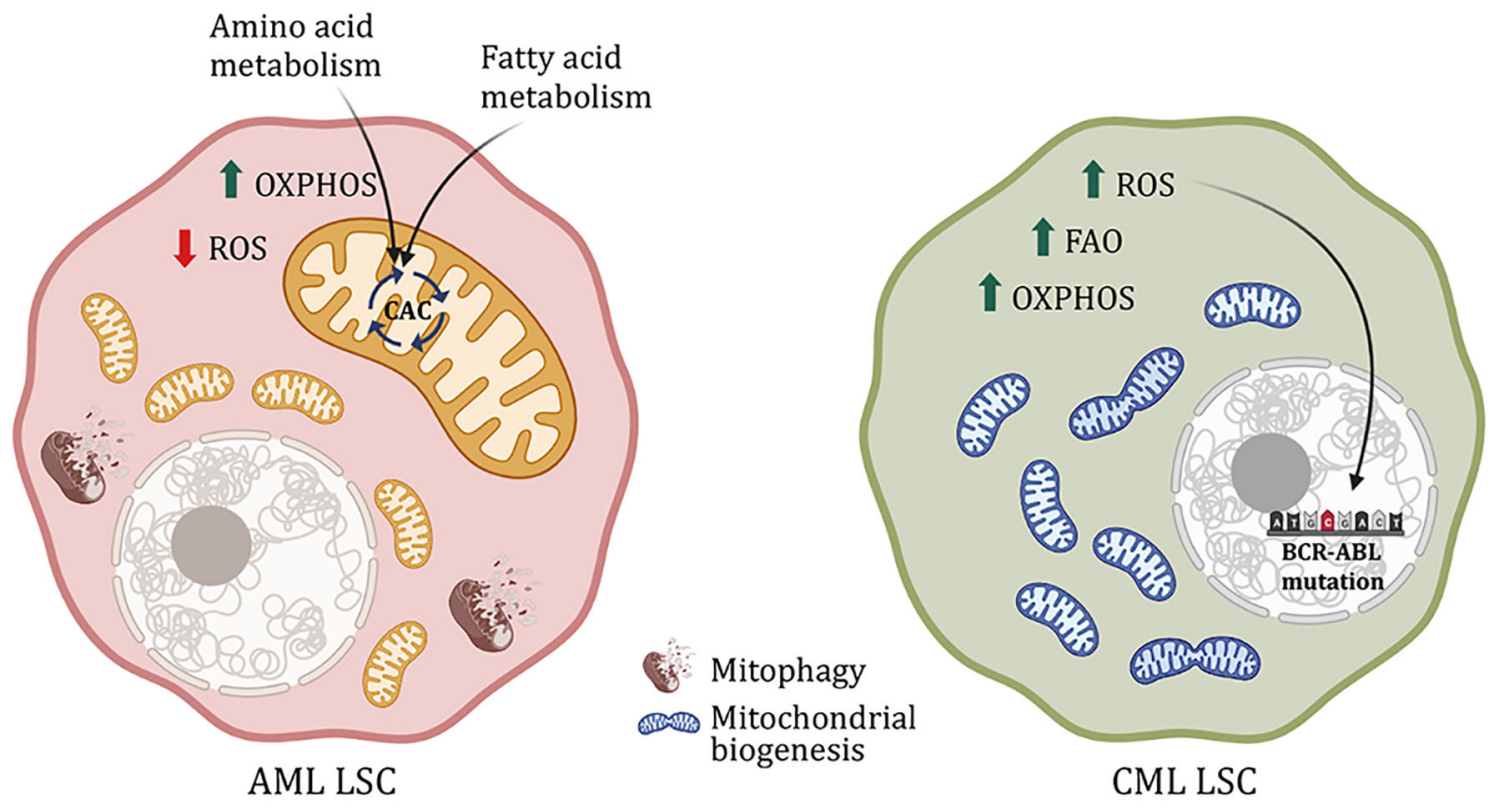
Metabolic difference between AML and CML LSCs. AML LSCs are reliant on OxPhos and yet have low ROS level controlled by mitophagy. Moreover, AML LSCs use amino acid and fatty acid metabolism to fuel the citric acid cycle. CML LSCs on the other hand exhibit increased mitochondrial biogenesis, FAO, OXPHOS and ROS. ROS leads to DNA instability and subsequently. Causes mutation in the BCR-ABL oncogene, rendering the cells resistant to drug treatment.

Dependency of AML LSCs on mitochondrial function is associated with decreased levels of mitochondrial reactive oxygen species and concomitantly increased reliance on OxPhos rather than glycolysis as in normal HSCs to meet the cell’s energy demand (25). Indeed, instead of relying on incomplete glucose oxidation through the Embden-Meyerhof-Parnas glycolytic pathway, AML LSCs depend on substrate oxidation – especially fatty acid and amino acid catabolism – in mitochondria. One potential reason could be increased expression of pyruvate dehydrogenase kinase (PDK) observed in AML patients, which inhibits glycolysis by phosphorylating and inactivating the enzyme pyruvate dehydrogenase (29, 30). Inhibiting amino acid metabolism and FAO – especially very-long-chain, polyunsaturated fatty acids - along with the traditional therapy eradicates the sensitive and resistant AML LSCs, respectively (31–36). More specifically, non-essential amino acid cysteine, which forms an integral part of glutathione synthase functionality as the rate-limiting substrate for glutathione biosynthesis, maintains the redox balance in AML and prevents oxidative stress (**Figure 2**). Inhibition of cysteine or depletion of glutathione impairs the activity of electron complex II, subsequently inhibiting OxPhos, eradicating the AML LSCs (37, 38). Glutamine is another amino acid that feeds into the CAC and regulates OxPhos. Inhibition of glutaminase, a critical enzyme for glutamine metabolism, has also been found to eliminate AML LSCs (39–41). Glutaminolysis also fuels the synthesis of glutathione – the main soluble antioxidant metabolite - by providing one of the rate-limiting substrates along with cysteine – glutamate (42–44). Regulation of glutathione synthesis by a mechanism of cysteine depletion or inhibition of glutaminolysis is in part controlled by sirtuin 5 (SIRT5) and is a targetable metabolic vulnerability in AML (38, 45). Glutathione synthesis and recycling are also influenced by the kinase ataxia telangiectasia mutated (ATM) through transcriptional control of glucose 6-phosphate dehydrogenase (G6PD), the rate-limiting enzyme of the pentose phosphate pathway (PPP) (46). PPP generates reducing equivalent (the reduced form of nicotinamide adenine dinucleotide phosphate - NADPH) essential to the recycling of oxidized glutathione, and rate-limiting cofactors for anabolic reactions, like fatty acid and cholesterol synthesis. The availability of NADPH, and its non-phosphorylated precursor NAD (critical for metabolic reactions in glycolysis and CAC/OxPhos), is also constrained by NAD synthesis, which can be pharmacologically targeted in AML (43). Both ATM and G6PD are targetable to sensitize AML cells to chemotherapy (47). Another alternate glucose metabolism pathway, the hexosamine biosynthesis pathway (HBP), is also upregulated in AML patient samples. Inhibition of glutamine fructose-6-phosphate amidotransferase (GFAT), a rate-limiting enzyme of HBP, induces differentiation and apoptosis of AML cells and eliminates the tumor burden not only from the bone marrow but also the peripheral blood in AML xenograft mouse models (48). Although extensive studies have been done to understand metabolism in AML and drug-resistant AML, it is still unknown whether these metabolic changes are common across all AML sub-types or whether the oncogene plays a role in deciding the metabolic fate of the AML cells, both the blast and LSCs.

### Chronic Myeloid Leukemia (CML)

CML is more prevalent in the geriatric population. Due to a single translocation in phenotypic HSCs, it forms BCR-ABL, a constitutively active tyrosine kinase. Unlike normal HSCs, CML cells are highly dependent on OxPhos regulated by sirtuin 1 (SIRT1)-mediated activation of peroxisome proliferator-activated receptor γ coactivator 1α (PGC1α) (49–51). SIRT1 is a NAD-dependent histone deacetylase that deacetylates and activates PGC1α, a regulator of mitochondrial biogenesis, both of which are found upregulated in CML. Dual treatment with SIRT1 and tyrosine kinase inhibitors (TKI) reduces CML cell proliferation, mitochondrial gene suppression and subsequent cell death in transgenic CML mouse model (51). Additionally, as opposed to AML, in CML, an increase in OxPhos also increases ROS production, causing DNA damage and genomic instability. This leads to mutations in the BCR-ABL oncogene, eventually causing oncogene-dependent resistance to TKI (**Figure 2**) (52–54). With TKI treatment, CML stem and progenitor cells accumulate lipids as well as nucleic acids with an increase in FAO (55). Moreover, metabolic stress created by TKI activates AMPK, suppressing which reduces disease progression (56). AMPK regulates energy homeostasis and leads to glucose and fatty acid uptake upon activation. And in the case of BCR-ABL-independent TKI resistance, glycolytic genes are upregulated along with increased glucose uptake, lactate production, and reduced oxygen consumption (50, 57–59). Although, this metabolic phenotype is partly similar to normal HSCs, the difference between HSCs and resistant CML LSCs is the dependency on the glycolytic pathway. Inhibition of PKM2 and LDHA, important enzymes for aerobic glycolysis, reduces disease progression and improves survival of CML mice, without impacting the surrounding HSCs (4, 59, 60). On the other hand, in advanced blast crisis CML, the stem cells have increased branched-chain amino acid metabolism (61). Additionally, these cells also upregulate CD36 fatty acid transporter and utilize gonadal adipose tissue lipolysis to fuel fatty acid metabolism as a source of energy as well as evade chemotherapy (62). Hence, targeting mitochondrial metabolic pathways in the CML stem and progenitor cells along with TKI treatment increases cell death and improves survival (49, 58, 63).

## Metabolism in Lymphoid Leukemia

### Acute Lymphocytic Leukemia (ALL)

**Figure 3:**
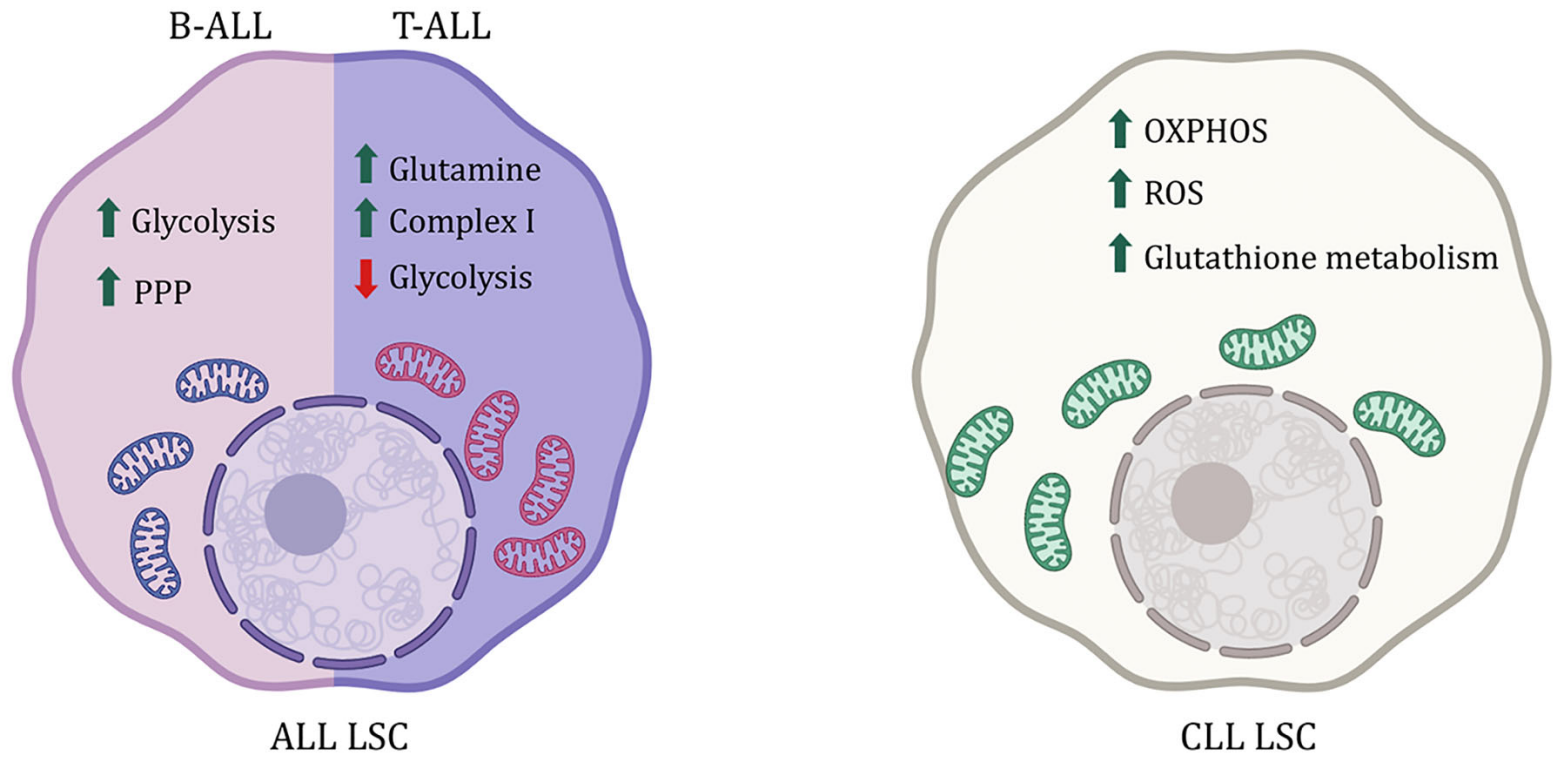
Metabolic difference between ALL and CLL LSCs. B-ALL LSCs are reliant on Pentose phosphate pathway (PPP) and glycolysis where as T-ALL LSCs rely more on glutamine metabolism. CLL on the other had sees an increase in ROS and is reliant on OXPHOS. Increase in glutathione metabolism is also reported as an antioxidant to the high amount of ROS.

ALL is the most common childhood leukemia marked by accumulation of immature B-cells (B-ALL) or T-cells (T-ALL). Chromosomal abnormalities like BCR-ABL translocation in the lymphoid progenitor lead to B-ALL, while T-ALL occurs due to gain-of-function Notch1 mutation in the lymphoid progenitor cells (64). B-ALL CD34+ cells from human BM have an upregulation of genes regulating glycolysis like glucose transporters *Glut1*, *Glut4*, and *Ldh*, with downregulation of CAC and FAO-related genes like *Idh3b*, *Sdhc*, *Fh*, and *Mdh* (65). They have increased glucose consumption and lactate production compared to the non-transformed human CD34+ cells indicating utilization of aerobic glycolysis in the former (65, 66). Additionally, increased glucose consumption in B-ALL cells and glutamine metabolism are linked to therapy resistance (67–69). Inhibiting glycolysis and glucose uptake reduced nucleotide and amino acid metabolism, decreased leukemogenesis and proliferation, increased apoptosis of B-ALL cells and sensitized these cells to glucocorticoid treatment (66, 68). Moreover, glucose is utilized not only for glycolysis but also as a starting point for PPP to reduce oxidative stress. B-cell genes *Pax5* and *Ikzf1* usually repress *G6pd*, a rate-limiting enzyme of PPP, hence reducing PPP activity (**Figure 3**). However, in B-ALL, the enzyme PP2A (Protein Phosphatase 2) switches glucose utilization from glycolysis to PPP while glycolysis is predominant HSCs. Inhibiting PP2A as well as G6PD or activating *Pax5* and *Ikzf* leads to reduced PPP activity sensitizing B-ALL cells to treatment (70). This target’s the unique vulnerability of B-ALL cells without impacting the non-transformed cells. Glucose utilization is important for the survival of B-ALL cells, whereas NOTCH1 activation in T-ALL leads to a metabolic switch from glycolysis to glutaminolysis (71, 72). The reduced glycolysis in T-ALL cells compared to normal T-cells can be attributed to Notch1-mediated AMPK activation (73). Moreover, inhibition of Notch1 signaling also leads to accumulation of glutamine and increased activity of complex 1 in the mitochondrial electron transport chain, conferring resistance to treatment (**Figure 3**) (72). Inhibition of glutamine synthesis as well as loss of AMPK signaling along with Notch1 inhibition sensitizes T-ALL cells towards apoptosis (72, 73). In addition to glucose and glutamine metabolism, targeting one carbon metabolism through the inhibition of serine hydroxymethyltransferase (SHMT) interferes with the supply of NADPH and one-carbon pools for proliferation has shown promise as a therapeutic strategy in treating T-ALL. In line with these findings, the amount of dietary folate has been shown to modulate metabolism in hematopoietic cells (74, 75). This signifies that depending on the type of ALL cells rely on different metabolic pathways.

### Chronic Lymphocytic Leukemia (CLL)

CLL like CML is found mainly in older adults marked by mutated mature CD5+ B-cells or memory B-cells in BM, blood, and even lymph nodes (76). Resting B-cells and memory B-cells are more glycolytic than activated B-cells (77). However, in CLL, the mutated B-cells rely on OxPhos over glycolysis and have reduced glucose uptake unlike normal HSCs (78, 79). CLL lymphocytes have an increase in ROS plus mitochondrial respiration and also have an active antioxidant activity *via* glutathione metabolism, protecting CLL cells from chemotherapy (**Figure 3**) (80, 81). In line with this, CLL lymphocytes also have overexpressed glutamine dehydrogenase, which plays a role in glutathione synthesis, and abolishing the glutathione-mediated protection mechanism leads to apoptosis of CLL cells (81, 82). Moreover, poor prognosis in CLL is marked by an accumulation of lipids, particularly ceramide and lipoprotein lipases, indicating active lipid metabolism, making them susceptible to FAO inhibitors, even in cases of treatment resistance (83–87).

Overall, one can affirm that metabolism is plastic, and different leukemia types have various metabolic vulnerabilities. Moreover, metabolism also changes with drug treatment. Understanding the mechanisms of metabolic change in leukemia, and following drug treatment, is important to identify target pathways without impacting the normal cells.

## Limitations and Advancements

### Cell Number

Cell number limitation is one of the major impediments to understanding metabolic differences in the cells of interest, such as LSC. Due to the need for millions of cells for metabolic assays, the studies are usually conducted with a broader pool of cells or cell lines instead of the specific cells of interest in a complex model. Furthermore, metabolic differences due to phenotypic cellular heterogeneity, even within genetically homogeneous cell populations should be considered. All of this makes it challenging to have conclusive results for the cell of interest. This highlights the need to improve metabolic techniques to study smaller numbers of cells. One such advancement has been in metabolic profiles using ultra-high pressure liquid chromatography-mass spectrometry (UHPLC-MS). A high-throughput UHPLC-MS method developed recently can profile metabolites from as few as 10,000 cells (59, 88–90). The technique involves the use of a larger pool of cells from the same tissue and of heavy labeled standard spike-in to call peaks while analyzing the few cell samples (59, 88). Additional methods are being developed to afford the quantitation of small molecule metabolites from hundreds of cells at the expense of the breadth of the coverage of multiple metabolic pathways (18, 91). More targeted, single-cell metabolomics approaches have also been proposed, which leverage CyTOF (cytometry of time of flight)- or capillary electrophoresis (CE)-based MS techniques for rapidly detecting a subset of metabolites (92). This technique allows to determine metabolite abundance in a specific population pool where cell number is a limitation, for instance, HSCs and LSCs (9, 31, 89, 93). Global metabolic profiling at steady state provides a snapshot view of the abundance of metabolites in cells. However, it fails to determine whether metabolites accumulate because of increased production or reduced usage. Thus, to infer the activity of a specific metabolic pathway, UHPLC-MS needs to be accompanied by other metabolic assays.

### *In Vivo* Metabolic Flux

To understand the metabolic pathway activity over time and in different conditions, metabolic flux analysis provides more detailed information about substrate preference and consumption rate for catabolic or anabolic purposes. Metabolic tracing with stable isotope-labeled substrates has been widely utilized but more in cell lines which are usually not representative of the complexity of the cells within their environment. *In vivo* metabolic flux analysis (especially with radiolabeled or fluorescent tracers), on the other hand, proves efficient in such cases (94, 95). The most widely used methods to administer heavy carbon labeled tracers in a mouse are by gastric gavage or bolus injection *via* a tail vein or continuous infusion *via* cannulation (96). These administration methods can achieve a high plasma concentration of the tracer; however, they also cause stress to the animal due to anesthesia or animal handling, defeating the purpose of studying metabolism at steady state (97). To overcome this limitation and enable long-term tracer administration, tracers can also be delivered through water (97). Although this technique reduces stress in mice, facilitating studies at steady state, it fails to consider the variability in the diet among individual mice and hence the variability of the amount of tracer administered. Additionally, the possible metabolism of tracer as it circulates through the hepatic portal system and before it reaches the organ of interest must be taken into consideration. Even though metabolic flux assays provide more information than global metabolic profiles, the limitation to these methods is the time window available to harvest the tissue and cells of interest without losing the cellular tracing. For this reason, researchers flash-freeze the whole organ soon after harvesting. But this limits the study of the metabolic flux to an organ as a whole instead of individual cell types within the organ. The field needs a better technique to understand metabolic fluxes at the cellular level, and this is one of the reasons why *in vivo* metabolic tracing of bone marrow has not been carried out until very recently (98).

### Metabolic Heterogeneity

Metabolic heterogeneity between tissues has been widely studied; however, it is yet uncovered between cells within the same tissue and between tumors (96). One major impediment in analyzing metabolites at a single cell level is the sensitivity of metabolite detection. Metabolite reporter using an alkyne tagged ‘Click’ chemistry is a potential solution to overcome this limitation (99). Using different alkyl groups can aid in multiplexing different samples and facilitate the use of multiple tracers in the same experiment, thus saving cost and time (99). Moreover, the enhanced signal of alkyne detection by MS helps determine metabolites at a single-cell level (99). However, it becomes difficult to identify one cell especially with overlapping peaks (99). In the current era of single-cell analysis, a computational model for studying metabolic phenotypes in the tumor microenvironment at a single cell level has also been developed (100), along with methods for imaging single-cell behavior in various microenvironments (101). They combine global metabolic gene expression with dimensional reductionists model and clustering algorithm to determine the metabolic gene expression profile of individual cells in head and neck tumors and melanoma (100). Although this technique considers heterogeneity and depicts metabolic profiles of different cell types, gene expression cannot always be correlated to metabolic activity. Further metabolic enzyme-based assays should be carried out to validate the computational analysis.

Some of the widely used techniques to determine heterogeneity based on mitochondrial mass and activity involve electron microscopy and confocal imaging using fluorescent markers like mitotrackers for mitochondrial abundance in each cell, TMRE for determination of mitochondrial membrane potential, or even fluorescent labeling of metabolic enzymes. However, these *ex vivo* fluorescence markers do not yield reproducible information. The results vary with changes in concentration, temperature, oxygen concentration, and cell count, pointing towards the importance of detailed experimental descriptions in publications. One recent imaging technique developed to measure mitophagy *in vivo* is based on a transgenic mouse model expressing the pH-sensitive fluorescent protein mt-Kiema localized to the mitochondria (102). Upon mitophagy, the pH of the mitochondria drops, changing the color of the mt-Kiema protein (102). Crossing these mice to transgenic leukemic mice would help expand our understanding of mitophagy in normal HSCs versus LSCs with or without drug treatment. This model can also be used for time-lapse studies as well as live-cell imaging. Another technique to study metabolic heterogeneity, called fluorescence-activated mitochondria sorting (FAMS), is based on flow cytometry. Using this technique, mitochondria from different types of tissues can be isolated based on their membrane potential, mitochondrial protein markers and (103).

Currently, no technique alone can be used to understand the metabolic complexity of a heterogeneous cell population. Therefore, it is important to use different metabolic assays to address the same question before making a conclusive statement.

### Drug Targeting

Understanding metabolic differences in tumor versus normal cells has recently seen significant advancements. Many drugs have been repurposed as metabolic inhibitors or activators, like tigecycline (a known antibiotic) which can inhibit mitochondrial protein translation leading to inhibition of OxPhos (49, 104). Another drug repurposed is metformin (clinically used as an anti-diabetic) which is being used as an AMPK activator, currently in phase II clinical trials for CLL (ClinicalTrials.gov: NCT01750567) (105, 106).

However, most of these drugs are non-specific or have multiple targets; for instance, a widely used AMPK inhibitor, dorsomorphin, can also inhibit VEGF and BMP-SMAD signaling (107), while shikonin, a drug used for PKM2 inhibition, can also inhibit STAT3, Fak, Src, cMyc and PI3K-AKT signaling pathways and cause cellular apoptosis *via* activation of c-JUN-N-terminal kinase (JNK) (108–111). Another example would be mubritinib, a known inhibitor of ERRB2, that can localize to the mitochondria and inhibit complex I of the electron complex chain (112). New metabolic inhibitors are being developed, for instance, IACS-010750, an inhibitor of elector complex chain complex 1, a drug currently under phase I clinical trials for AML (ClinicalTrials.gov: NCT02882321) (113). To keep up with the metabolic discoveries, inhibitors with more specificity need to be developed to reduce side effects and off-target impacts and provide better therapeutic options.

## Conclusion

Metabolism can still be considered a developing field of cellular biology. No single technique can or should be used to address questions regarding stem cell metabolism. With the advent of single-cell technologies for transcriptomic and epigenetics, science has moved from bulk cellular analysis to understanding cellular heterogeneity within the same population, which needs to be adapted even in metabolism. Moreover, the changes in metabolism are not unique to stem cells, highlighting the need for studies to be carried out with appropriate controls to enhance LSC specificity and reduce side effects of metabolic inhibitors on HSCs. These metabolic differences would represent a condition of LSC-specific vulnerability that could potentially be targeted.

## Author Contributions

SP outlined and wrote the manuscript; TN and AD provided intellectual help with manuscript writing; RW supervised and assisted with manuscript writing. All authors contributed to manuscript revision, read, and approved the submitted version.

## Funding

This project was supported by NIH grants 1PO1HL131477; startup funds from the Division of Hematology/Oncology at the University of Alabama at Birmingham (UAB); American Cancer Society-IRG Junior Faculty Development Grant (2019); the American Society of Hematology Bridge Grant (2018); and the Leukemia Research Funding (2019).

## Conflict of Interest

The authors declare that the research was conducted in the absence of any commercial or financial relationships that could be construed as a potential conflict of interest.

## Publisher’s Note

All claims expressed in this article are solely those of the authors and do not necessarily represent those of their affiliated organizations, or those of the publisher, the editors and the reviewers. Any product that may be evaluated in this article, or claim that may be made by its manufacturer, is not guaranteed or endorsed by the publisher.
